# A Novel Prognostic Marker in Severe Traumatic Brain Injury Patients: Pbto2/Pao2 Ratio

**DOI:** 10.1186/2197-425X-3-S1-A487

**Published:** 2015-10-01

**Authors:** R Badenes, A Lozano, F Bilotta, A Cueva, B Ortolá, A Maruenda, J Belda

**Affiliations:** Hospital Clinico Universitario de Valencia, Anesthesiology and Surgical-Trauma Intensive Care, Valencia, Spain; Hospital Clinico Universitario de Valencia, Valencia, Spain; Sapienza University of Rome, Rome, Italy; Hospital de la Ribera, Alzira, Spain

## Introduction

Traumatic brain injury (TBI) is a common cause of morbidity and mortality worldwide. Neuromonitoring has evolved considerably in recent years in TBI patients. Utilization of brain tissue oxygenation (pBtO2) is an important variable in the treatment of traumatic brain injury.

## Objectives

The main goal is to examine whether clinical scales (Glasgow Coma Scale (GCS), Injury Severity Score (ISS), radiographic scales based on admission computed tomography (Marshall), pBtO2 and pBtO2/paO2 ratio are associated with clinical outcome after severe TBI.

## Methods

This is a prospective observational study with institutional review board approval. Patients TBI and a Glasgow Coma Scale score ≤8 were identified on admission. Glasgow Coma Scale (GCS), Injury Severity Score (ISS), radiographic scales based on admission computed tomography (Marshall), IntraCranial Pressure (ICP), pBtO2 and pBtO2/paO2 during first 24 hours were recorded. Patient outcome was determined as favorable (patient had no, little, or moderate disability) or unfavorable (patient died, was in a vegetative state or had severe disability) using the Glasgow Outcome Scale by medical record review at 6 months after TBI. We considered an ordinal analysis with proportional odds methodology. a logistic regression model was used to calculate the 95% CI for the odds of unfavorable outcome.

## Results

Over a 3-year period, 32 patients were entered into the study. the mean age was 37 ± 17.4 years, injury severity score was 27.7 ± 10.7, and Glasgow Coma Scale score was 5.2 ± 3.4. At 6 months, 7 (21.8%) patients had died, and 20 (59.3%) had a favorable outcome. Favorable outcome consistently demonstrated higher pBtO2/paO2 ratio compared with nonsurvivors including age as a covariate(p < 0.001). Factors associated with unfavorable outcome (univariate analysis): ISS>50 (OR 1.47, CI 1.22-1.68), Marshall ≥ 3 (OR 1.88, CI 1.44-2.20), ICP > 20 (OR 2.88, CI 2.52-3.24), GCS < 5 (OR 2.11, CI 1.94-2.30), PtiO2 < 20 (OR 2.16, CI 2.05-2.27) and pBtO2/paO2 ratio < 0.2 (OR 3.88, CI 3.48-4.27).

## Conclusions

The first 24 hours of pBtO2/paO2 ratio < 0.2 predicts mortality and outcome. When pBtO2/paO2 ratio remains below 0.2 in the first 24 hours of monitoring, mortality and unfavorable outcome is increased. This study challenges the brain oxygenation threshold of 20 mm Hg that has been used conventionally and delineates a time for monitoring pBtO2/paO2 ratio that is predictive of outcome.
